# Robotic Kinematic measures of the arm in chronic Stroke: part 2 – strong correlation with clinical outcome measures

**DOI:** 10.1186/s42234-021-00082-8

**Published:** 2021-12-29

**Authors:** Caio B. Moretti, Taya Hamilton, Dylan J. Edwards, Avrielle Rykman Peltz, Johanna L. Chang, Mar Cortes, Alexandre C. B. Delbe, Bruce T. Volpe, Hermano I. Krebs

**Affiliations:** 1 Department of Mechanical Engineering, Massachusetts Institute of Technology, 77 Massachusetts Avenue, Cambridge, MA 02139 USA; 2 Universidade de Sao Paulo, Avenida Trabalhador Saocarlense – 400, Sao Carlos, SP Brazil; 3 Moss Rehabilitation Research Institute, 60 Township Line Rd, Elkins Park, PA 19027 USA; 4 Rehabologym, 90 N Broadway, Irvington, NY 10533 USA; 5 Feinstein Institute for Medical Research, 350 Community Dr, Manhasset, NY 11030 USA; 6 Icahn School of Medicine at Mount Sinai, 1 Gustave L. Levy Pl, New York, NY 10029 USA

**Keywords:** Stroke, Kinematics, Outcome measures, Correlation, Robotics, tDCS

## Abstract

**Background:**

A detailed sensorimotor evaluation is essential in planning effective, individualized therapy post-stroke. Robotic kinematic assay may offer better accuracy and resolution to understand stroke recovery. Here we investigate the added value of distal wrist measurement to a proximal robotic kinematic assay to improve its correlation with clinical upper extremity measures in chronic stroke. Secondly, we compare linear and nonlinear regression models.

**Methods:**

Data was sourced from a multicenter randomized controlled trial conducted from 2012 to 2016, investigating the combined effect of robotic therapy and transcranial direct current stimulation (tDCS). 24 kinematic metrics were derived from 4 shoulder-elbow tasks and 35 metrics from 3 wrist and forearm evaluation tasks. A correlation-based feature selection was performed, keeping only features substantially correlated with the target attribute (R > 0.5.) Nonlinear models took the form of a multilayer perceptron neural network: one hidden layer and one linear output.

**Results:**

Shoulder-elbow metrics showed a significant correlation with the Fugl Meyer Assessment (upper extremity, FMA-UE), with a R = 0.82 (*P* < 0.001) for the linear model and R = 0.88 (*P* < 0.001) for the nonlinear model. Similarly, a high correlation was found for wrist kinematics and the FMA-UE (R = 0.91 (*P* < 0.001) and R = 0.92 (*P* < 0.001) for the linear and nonlinear model respectively). The combined analysis produced a correlation of R = 0.91 (*P* < 0.001) for the linear model and R = 0.91 (*P* < 0.001) for the nonlinear model.

**Conclusions:**

Distal wrist kinematics were highly correlated to clinical outcomes, warranting future investigation to explore our nonlinear wrist model with acute or subacute stroke populations.

**Trial registration:**

http://www.clinicaltrials.gov. Actual study start date September 2012. First registered on 15 November 2012. Retrospectively registered. Unique identifiers: NCT01726673 and NCT03562663.

**Supplementary Information:**

The online version contains supplementary material available at 10.1186/s42234-021-00082-8.

## Background

Despite significant medical advances in acute management over the last 20 years, stroke continues to be a leading cause of chronic disability and health-related costs in the United States (Lopez et al., 2006) and across the globe (Jauch et al., 2013). Following the substantial progress in stroke-survival rates, there are now more people living with chronic stroke-related disability, impacting the health burden (Murray et al., 2013). Limited functional recovery of the upper extremity (UE) is often reported following stroke and is the cause of significant long-term restriction to activities of daily living (ADLs) and community reintegration (Lawrence et al., 2001). More standardized and targeted measures of UE function are key to prescribing more effective and individualized therapy, with the aim of improving patient outcomes and reducing length of stay, the costs, and the demand for rehabilitation following stroke (Bernhardt et al., 2019; Harcum et al., 2019).

A detailed evaluation of sensorimotor impairment and function is essential in planning appropriate, effective, and individualized therapy interventions (Harcum et al., 2019; Rech et al., 2020). Clinical outcome measures, such as the widely used Fugl-Meyer Assessment of Upper Extremity Motor Recovery after Stroke (FMA-UE), offer a convenient and low cost method of assessment. These measures have been used as the gold standard for evaluation of rehabilitation outcomes across the continuum of care and in clinical research. Nonetheless, many clinical scales are limited by subjectivity and insensitivity to detect changes in motor control, particularly for patients with severe motor impairment (Krebs et al., 2002; Bosecker et al., 2010; Semrau et al., 2013; See et al., 2013). Even for highly recommended clinical measures with good psychometric properties, such as the FMA-UE, previous studies have highlighted that without adequate training and standardization of assessment procedures, the variability in inter-rater scores can be equivalent to the scale’s minimally clinically important difference (See et al., 2013; Krebs et al., 2014). It is not well understood that the FMA-UE is a nonlinear scale, meaning gains on the items at the beginning of the measure are not equivalent, and have a different meaning, to items at the end of the measure. There are also varied reports and uncertainty regarding the extent that the FMA-UE scale can stratify patients at different stages of recovery and the impact of ceiling and floor effects (See et al., 2013; Gladstone et al., 2002; Cramer, 2010). With rapid advances and improved accessibility of rehabilitation technology in the developed world, the reliability of clinical measures is now being questioned given that other measurement tools are more available.

Robotic derived kinematic evaluations may be one solution to better understand stroke recovery and optimize patient’s rehabilitation journey. The ability to objectively evaluate movement quality is key for distinguishing between behavioral restoration and compensation post-stroke. (Kwakkel et al., 2019) Previous studies in this field suggest that kinematic data can provide a standardized and objective measure of a patient’s motor control and movement quality, which correlates with well-known clinical measures, (Bosecker et al., 2010; Semrau et al., 2013; Krebs et al., 2014; Colombo et al., 2005; Dipietro et al., 2012; Dukelow, 2017; Agrafiotis et al., 2021) and has the potential to enhance knowledge of treatment effects, clinical reasoning, and our understanding of stroke recovery (Bernhardt et al., 2019; Kwakkel et al., 2019). The existing literature on correlating robotic kinematic data with clinical measures has been limited by small sample sizes, (Zollo et al., 2011; Murphy et al., 2011; Krabben et al., 2012) enrolling patients with a narrow spectrum of UE impairment, (Zollo et al., 2011; Murphy et al., 2011) only including data on the proximal UE, (Bosecker et al., 2010) or investigating a single modelling technique, (Bosecker et al., 2010; Krebs et al., 2014; Agrafiotis et al., 2021) potentially impacting the perceived value and strength of the correlation between robotic kinematic evaluations and clinical scales.

This study aims to contribute to this field in two ways. First by investigating the value of kinematic wrist data (in addition to the previously studied shoulder-elbow metrics) to improve the correlation between standardized robotic measures and common clinical measures of the UE. Secondly, we will build on previous work (Bosecker et al., 2010; Krebs et al., 2014; Agrafiotis et al., 2021) by investigating both linear and nonlinear regression models for estimating clinical measures from robot metrics in the chronic post-stroke population.

## Methods

### Study overview

This study was derived from a randomized controlled trial conducted from 2012 to 2016 investigating the combined effect of intensive robotic therapy and transcranial direct current stimulation (tDCS). Subjects were enrolled from 2 sites, Burke Neurological Institute and Feinstein Institute for Medical Research, to participate in a double-blind, sham-controlled, repeated measures study. Candidates were eligible if they presented with chronic stroke (> 6-months when commencing the intervention) and right hemiparesis. The robotic intervention involved 1024 movement repetitions per session, alternating shoulder-elbow (MIT-MANUS, planar robot) and wrist-forearm robot therapy (MIT-WRIST, a 3 degree of freedom wrist robot) on separate days, performed 3 times a week (for a total of 36 sessions) (Krebs et al., 2007; Krebs et al., 1998). Both the Robot_tDCS_ (anodal-tDCS, 2 mA, affected hemisphere, M1/SO montage) and Robot_Sham_ interventions were administered at rest for 20 min immediately prior to each robotic therapy session (Giacobbe et al., 2013). Baseline clinical and robotic evaluations were performed twice within 6-weeks prior to commencing the intervention (to ensure a stable baseline impairment), again at completion of the 12 week intervention, and after 6-months post training. Additional details of the study design are presented in Edwards et al. (2019).

### Clinical outcome measures for the UE

The primary clinical measure employed was the FMA-UE. The FMA-UE uses an ordinal performance-based scale to score the level of UE motor impairment after stroke. The scale comprises 33 items designed to examine reflex activity, motor control, and muscle strength (with a maximum score of 66). The measure is both highly recommended by stroke guidelines and widely used for clinical and research settings in chronic stroke (Shirley Ryan AbilityLab, 2016a).

Secondary outcome measures included the Wolf Motor Function Test (WMFT), Barthel Index (BI), and the Medical Research Council Motor Power score (MRC). The WMFT is a 21 item measure of UE motor ability comprising time, functional ability, and strength tasks. The maximum score is 75 with lower scores indicating lower functional ability. The WMFT is recommended for the assessment of stroke across the continuum of care (Shirley Ryan AbilityLab, 2016b). The BI is a performance measure of functional independence including mobility, gait, and performance of ADLs. 10 items are rated according to the amount of assistance required to successfully complete the task, with a maximum score of 100 (Shirley Ryan AbilityLab, 2016c). The MRC score is a grading system for manual muscle testing used widely in neurological and musculoskeletal clinical and research settings. The power of each muscle is evaluated in relation to the maximum expected for that single joint and scored from 0 to 5 (Shirley Ryan AbilityLab, 2020; Medical Research Council, 2020).

### Kinematic and kinetic measures of the UE

Twenty-four kinematic macro and micrometrics were derived from 4 shoulder-elbow evaluation tasks and 35 metrics from 3 wrist and forearm evaluation tasks. Details of the robotic kinematic tasks and metrics have been published elsewhere (Bosecker et al., 2010; Krebs et al., 1999) and are described in Tables 1 and 2. Figure 3 shows a representative example of three participants who were assessed at admission to have a low (severe impairment), or moderate FMA-UE score and their corresponding robotic evaluation movement plots at admission and follow-up.

**Table 1:** Outline of robotic evaluation tasks and the metrics derived from the evaluations

Robotic Evaluation	Description	Metrics Derived
Unconstrained trained reaching task (S/E robot)	Required the patient to attempt 80 active reaching motions to and from 8 targets spaced equally around a 14 cm circle. The reaching movements used for the evaluation were similar to the robot assisted tasks completed during training.	Aim, duration, deviation, dwell time, mean speed, peak speed, speed shape (ratio of mean to peak speed), and jerk (normalized for terminated reaching movements (discrete) and rhythmic movements.) Micrometric data was derived by extracting support-bounded lognormal submovements from movement speed profiles as described in Rohrer et al. (Rohrer et al., 2002) This included submovement number, duration, overlap, peak, and interpeak interval (see Table 2 for submovement definitions.)
Unconstrained trained wrist pointing task	Required the patient to attempt 80 active wrist (F/E/RD/UD) motions to and from 8 targets distributed around an ellipse with major axis of 60^o^ (30^o^ for F/E each) and minor axis of 30^o^ (15^o^ for RD/UD each). The wrist pointing movements used for the evaluation were similar to the robot assisted tasks completed during training.	Aim, duration, deviation, dwell time, mean speed, peak speed, speed shape (ratio of mean to peak speed), and jerk (normalized for terminated pointing movements (discrete) and rhythmic movements.) Micrometric data included submovement number, duration, overlap, peak, and interpeak interval (see Table 2 for submovement definitions.)
Unconstrained trained forearm movement	Required the patient to attempt 80 active forearm (PS) motions between 2 targets distributed along a line (30^o^ of P and S each). (Krebs et al., 2007) The forearm movements used for the evaluation were similar to the robot assisted tasks completed during training.	Aim, duration, deviation, dwell time, mean speed, peak speed, speed shape (ratio of mean to peak speed), and jerk (normalized for terminated pointing movements (discrete) and rhythmic movements.) Micrometric data included submovement number, duration, overlap, peak, and interpeak interval (see Table 2 for submovement definitions.)
Unconstrained untrained circle drawing task (S/E robot only)	Involved the patient completing 5 unassisted attempts to draw a circle, in a clockwise and counterclockwise direction, from 2 different starting positions (3 o’clock and 9 o’clock) for a total of 20 movement repetitions. Note that training did not include attempts to draw circles.	Major and minor axes of the best-fitting ellipse and the ratio of the axes measurements for each of the 4 circle drawing conditions as well as the orientation of the major axes. Inverse kinematics allow us to estimate the shoulder and elbow joint movements. Joint independence determines the correlation between the shoulder and elbow movement.
Movement against resistance task	Required the patient to move against an increasing force as they reach toward the targets.	Measures of maximum displacement and overall aim.
Isometric stabilization task	The patient attempted to hold their S/E or wrist still while the robot exerted forces to move the patient’s arm/robot handle toward the outer edge of the circle.	Movement scatter and offset.
Kinetic S/E evaluation	The patient was positioned facing the robot (for shoulder F/E) or rotated 90 degrees away from the robot (for shoulder AB/AD) in 90 degrees of shoulder flexion, with the elbow fully extended and the forearm, wrist, and hand supported by the robot arm. The patient was asked to attempt to lift their arm (for F and AB measurements) or push down (for E and AD measurements) 5 times in each direction, for a total of 20 trials.	Mean shoulder strength (deltaz)

Note: S/E = shoulder-elbow, F = flexion, E = extension, AB = abduction, AD = adduction, RD = radial deviation, UD = ulnar deviation, PS = forearm pronation and supination

**Table 2:** Description of submovement metrics

Submovement Metric	Definition
Number	The number of submovements in an entire movement
Duration (s)	The time from initiation until the termination of an individual submovement
Overlap (s)	Interval between commencement of a submovement and termination of the previous submovement
Peak (m/s (S/E) rad/s (wrist))	Peak speed of each individual submovement
Interpeak interval (s)	Interval between peaks of consecutive submovements

Note: S/E = shoulder-elbow. Metric definitions were adapted from Rohrer et al. (Rohrer et al., 2002)

### Statistical analyses

All statistical analyses were performed using MATLAB (Natick, MA, The Mathworks, Inc. vR2019b) and results were deemed significant if *P* values were < .05.

### Dataset format and model generation

A data analysis framework was developed to preprocess the robotic raw data (Moretti et al., 2020). This tool built on formerly developed and tested calculations (Bosecker et al., 2010; Dipietro et al., 2012) to automatically obtain the robotic kinematic and kinetic-based metrics of all study subjects.

The dataset format describes evaluation sessions (admission, discharge and follow-up) for all patients, where features (predictive attributes) are the mean-aggregated kinematic and kinetic values, with the respective clinical measures of a particular session set as the target attributes.

**Fig. 1:**
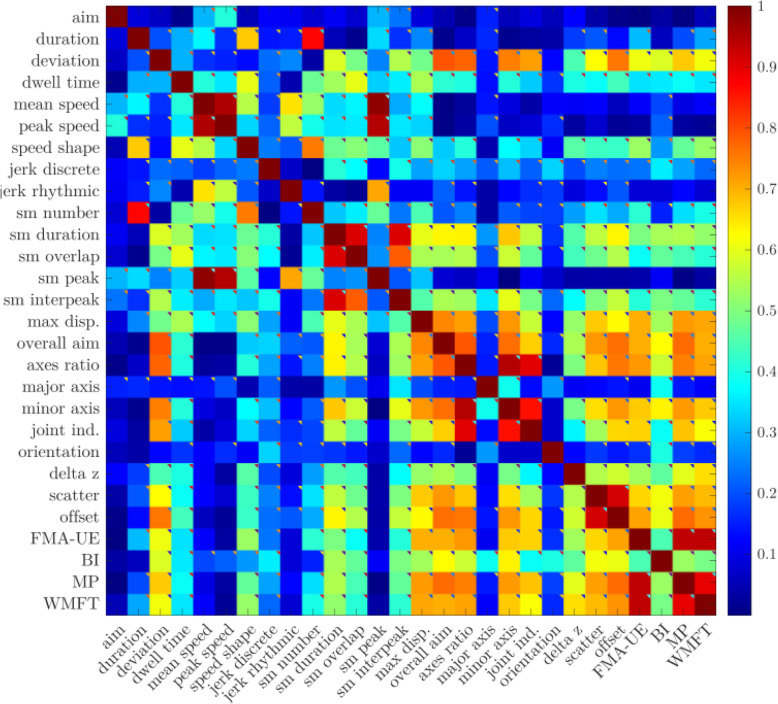
Heatmap of S/E kinematic and kinetic data correlation with clinical scales

**Fig. 2:**
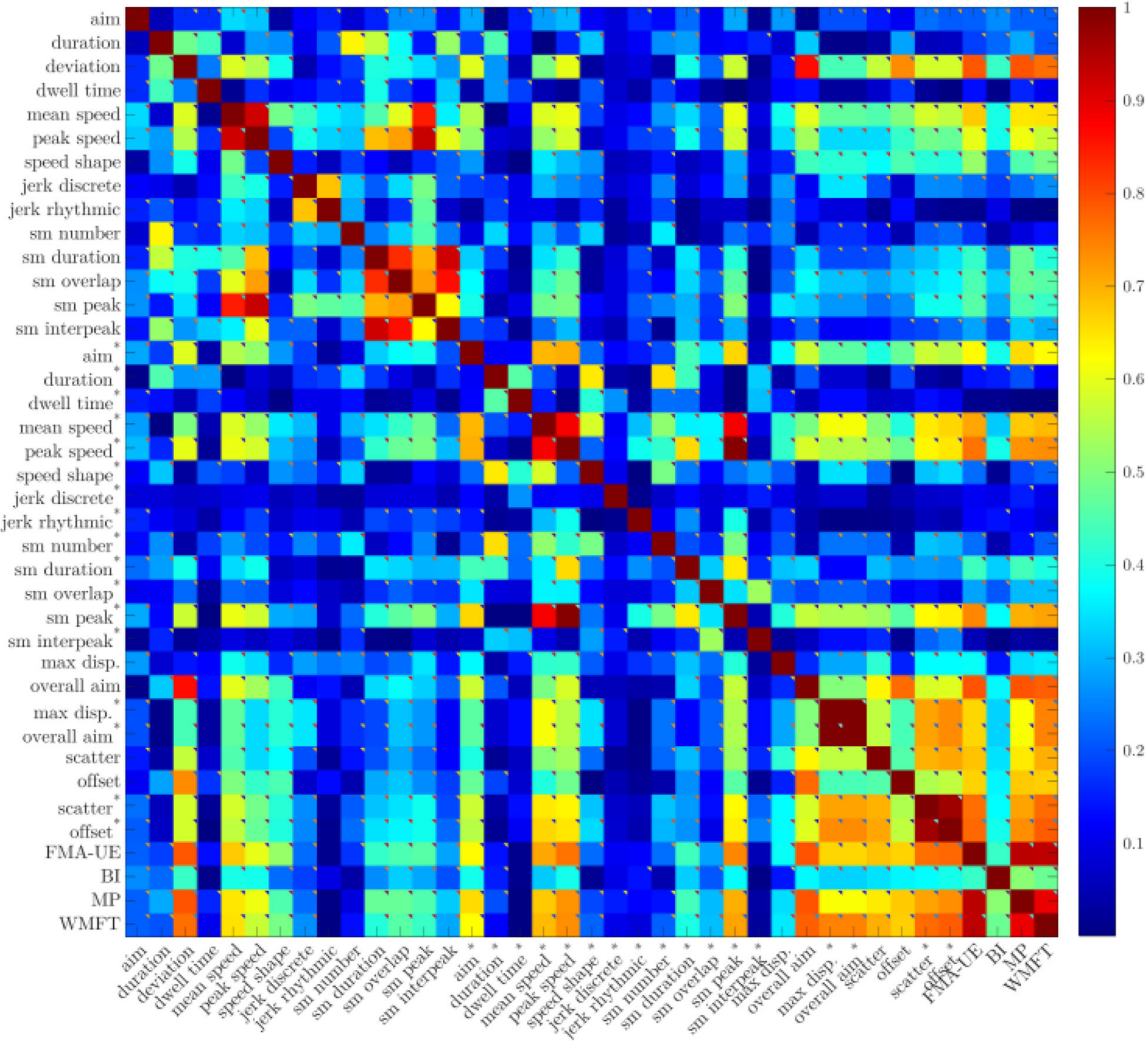
Heatmap of wrist-forearm kinematic data correlation with clinical scales

Due to high dimensionality of the dataset, we performed a correlation-based feature selection, keeping only features substantially correlated with the target attribute (R > .5, see Figs. 1 and 2) and representing the different motor control features (e.g. metrics extracted from both trained and untrained movements). In addition, for every group of features with high dependency among themselves, the one with better interpretability was preserved and the remaining features were discarded to reduce collinearity.

Similarly, correlations among the clinical measures were calculated to determine dependent scales (Figs. 1 and 2). If there was a very strong correlation between clinical measures, this suggested redundancy whereby it may be possible to reduce the number of clinical measures performed and the time needed to conduct a clinical evaluation.

The strength of the correlations calculated in the analyses (R) were interpreted as follows; R = .0–.3 very weak, R = .31–.5 weak, R = .51–.7 moderate, and R = .71–1.0 strong (Moore et al., 2013).

Following strategies from previous work, we considered linear (Bosecker et al., 2010) and nonlinear (Krebs et al., 2014; Agrafiotis et al., 2021) complexities as candidate models for the correlation of clinical scales across the duration of the study (i.e. two baselines, 12-week, and 6-months). Nonlinear models took the form of a multilayer perceptron neural network: one hidden layer with 2 sigmoid nodes and one linear output (Krebs et al., 2014; Agrafiotis et al., 2021) Features were rescaled by normalizing to their maximum values. Clinical measures were rescaled for the nonlinear model only.

Every candidate model was submitted to a 4-fold cross-validation, with the resulting R value representing the average. Because one patient was associated with more than one dataset instance (evaluation), we implemented a variation of a traditional cross-validation with patient-wise sampling instead of instance-wise. Instances from 25% of patients (randomly selected, without replacement) were held as a test set, while the remaining 75% were used to build a model for training. This process continued for each remaining 25–75% partition. A Mann-Whitney test was carried out to ensure that instances from training and test partitions were similarly distributed with respect to their expected outputs.

The shoulder-elbow motor power (MP) analysis was more complex and was performed in 3 ways to explore the correlation between the clinical MRC measure and the different kinematic and kinetic measures of shoulder force. The first correlation (MP total, Table 3) was calculated using kinetic metrics (derived from a force transducer) and all possible kinematic metrics (with a R > .5). The correlation analysis was repeated (labelled MP kinematic force metrics) using the kinetic metrics and only the force derived kinematic metrics (from the movement against resistance and isometric stabilization tasks) and finally, using only the kinetic metrics (MP force transducer metrics, Table 3) to determine the value of the different shoulder metrics (shoulder AB, AD, F, E, and mean shoulder force) and the use of a force transducer.

**Table 3:** Correlation of kinematic and kinetic metrics with clinical measures

Measure	Device	Model	R	*P*
FMA-UE 0–66	S/E	linear	0.82	**
		non linear	0.88	**
	wrist	linear	0.91	**
		non linear	0.92	**
	both	linear	0.91	**
		non linear	0.91	**
FMA-UE 1–38	S/E	linear	0.80	**
		non linear	0.86	**
	wrist	linear	0.86	**
		non linear	0.87	**
	both	linear	0.89	**
		non linear	0.89	**
FMA-UE 39–66	S/E	linear	0.74	**
		non linear	0.74	**
	wrist	linear	0.43	
		non linear	0.69	*
	both	linear	0.69	*
		non linear	0.70	*
BI	S/E	linear	0.68	**
		non linear	0.73	**
	wrist	linear	0.53	*
		non linear	0.58	*
	both	linear	0.67	**
		non linear	0.70	**
WMFT	S/E	linear	0.85	**
		non linear	0.89	**
	wrist	linear	0.89	**
		non linear	0.90	**
	both	linear	0.92	**
		non linear	0.93	**
MP (Total)	S/E	linear	0.86	**
		non linear	0.87	**
	wrist	linear	0.88	**
		non linear	0.90	**
	both	linear	0.91	**
		non linear	0.92	**
MP Kinematic Force Metrics	S/E	linear	0.86	**
		non linear	0.86	**
	wrist	linear	0.86	**
		non linear	0.90	**
	both	linear	0.90	**
		non linear	0.91	**
MP Force Transducer Metrics	S/E (combined mean force, deltaz)	linear	0.62	**
		non linear	0.66	**
	S/E (F/ E/ AB/ AD mean force)	linear	0.66	**
		non linear	0.69	**

Note: **P < 0.001, * *P* < 0.05. S/E = shoulder-elbow, FMA-UE = Fugl-Meyer Assessment of Upper Extremity Motor Recovery after Stroke, BI=Barthel Index, WMFT = Wolf Motor Function Test. MP (Total) = Motor Power, correlation analysis with the Medical Research Council Motor Power score (MRC) using both kinetic metrics and all possible kinematic metrics. MP Kinematic Force Metrics = correlation model using shoulder kinetic metrics and only force derived kinematic metrics (from the movement against resistance and isometric stabilization tasks). MP Force Transducer Metrics = using only the shoulder kinetic metrics with the MRC (mean shoulder force (deltaz) or individual mean measures of shoulder abduction (AB), adduction (AD), flexion (F), and extension (E))

## Results

### Subject demographics and baseline characteristics

Eighty-two participants were enrolled in the study in the chronic stage following stroke with a wide range of UE impairment. Table 4 summarizes the demographics of study participants and the admission scores for the clinical evaluations.

**Table 4:** Participant characteristics and admission results of clinical measures

Characteristic	Robot_**tDCS**_	Robot_**tDCS-Sham**_	Overall
Participants (n [%])	41 [50.0%]	41 [50.0%]	82 [100.0%]
Age (mean years, [range])	66.4 [42–87]	69.2 [42–90]	67.8 [42–90]
Days from stroke to start of trial (mean days [range])	1475.2 [226–6935]	1160.0 [151–6936]	1317.6 [151–6936]
Gender (n Female [%])	16 [39.0%]	16 [39.0%]	32 [39.0%]
Stroke location (n cortical [%])	26 [63.4%]	27 [65.9%]	53 [64.6%]
FMA-UE (mean [range])	25.6 [7–57]	25.4 [7–55]	25.5 [7–57]
WMFT (mean [range])	60.0 [1–169]	56.0 [0–167]	58.0 [0–169]
BI (mean [range])	88.4 [10–100]	85.0 [15–100]	86.7 [10–100]
MRC (mean [range])	46.8 [8–79.5]	44.1 [15–85]	45.5 [8–85]

Note: tDCS = transcranial direct current stimulation, FMA-UE = Fugl-Meyer Assessment of Upper Extremity Motor Recovery after Stroke, WMFT = Wolf Motor Function Test, BI=Barthel Index, MRC = Medical Research Council Motor Power score

### Correlation of robotic metrics and clinical scales

#### Correlation with FMA-UE

The 6 shoulder-elbow robotic metrics (features) with a R > .5 used for the correlation model with the FMA-UE included; offset, mean shoulder force (deltaz), circle joint independence, maximum displacement, deviation, and shape speed. Both the linear and nonlinear model fitted with these features showed a significant correlation with the FMA-UE, with a R = .82 (*P* < 0.001) for the linear model and R = .88 (*P* < 0.001) for the nonlinear model (see Table 3).

Five wrist and forearm metrics were selected for the correlation analysis; wrist deviation and mean speed, forearm mean speed, wrist offset, and forearm offset. A high and statistically significant correlation with the FMA-UE was calculated, with a R = .91 (*P* < 0.001) for the linear model and R = .92 (*P* < 0.001) for the nonlinear model.

The combined shoulder-elbow and wrist analysis (using the 6 shoulder-elbow and 5 wrist and forearm metrics) produced a correlation of R = .91 (*P* < 0.001) for the linear model and R = .91 (*P* < 0.001) for the nonlinear model.

#### Correlation with BI

Five shoulder-elbow metrics were found to have a R > .5; deviation and submovement duration, overall aim, horizontal axes, and offset. These shoulder-elbow features had a correlation of R = .68 (*P* < 0.001) using the linear model and R = .73 (*P* < 0.001) for the nonlinear model.

The 4 strongest wrist metrics were used for the BI correlation, although no metric was found to be R > .5; wrist deviation and mean speed, wrist overall aim, and forearm offset. The wrist correlations were lower, with R = .53 (*P* < 0.005) for the linear model and R = .58 (*P* < 0.005) for the nonlinear model.

Using the 9 shoulder-elbow, wrist, and forearm metrics, the combined analysis showed a moderate correlation of R = .67 (*P* < 0.001) for the linear model and R = .70 (*P* < 0.001) for the nonlinear model.

#### Correlation with WMFT

The same 6 shoulder-elbow and 5 wrist and forearm metrics as the FMA-UE correlation were found to have a R > .5 for the WMFT correlation analysis.

A strong correlation with the WMFT was found using the shoulder-elbow features for both the linear and nonlinear model (R = .85 (*P* < 0.001) and R = .89 (*P* < 0.001) respectively.)

The wrist also demonstrated a significant strong correlation with the linear and nonlinear models, with a R = .89 (*P* < 0.001) for the linear model and R = .90 (*P* < 0.001) for the nonlinear model.

The combined shoulder-elbow and wrist analysis resulted in a R = .92 (*P* < 0.001) for the linear model and R = .93 (*P* < 0.001) for the nonlinear model.

#### Correlation with MRC

##### MP Total

Five shoulder-elbow kinematic metrics and shoulder kinetic metrics were used in the shoulder-elbow MP total correlation analysis; offset, mean shoulder force (deltaz), circle joint independence, maximum displacement, and deviation. A significant correlation was calculated, with a R = .86 (*P* < 0.001) for the linear model and R = .87 (*P* < 0.001) for the nonlinear model.

The same 5 wrist and forearm metrics as the FMA-UE analysis were found to have a R > .5. The linear model had a R = .88 (*P* < 0.001) and the nonlinear model showed a R = .90 (*P* < 0.001).

The combined shoulder-elbow, wrist, and forearm analysis produced a R = .91 (*P* < 0.001) for the linear model and the nonlinear model showed a R = .91 (*P* < 0.001).

##### MP kinematic force

Out of the force derived shoulder-elbow kinematic metrics and shoulder kinetic metrics, 3 were found to have a R > .5; offset, mean shoulder force (deltaz), and maximum displacement. A significant correlation was found of R = .86 (*P* < 0.001) for both the linear and nonlinear models.

Four wrist and forearm force derived metrics were used for this analysis; both forearm and wrist offset, and wrist and forearm overall aim. The linear model showed a correlation of R = .86 (*P* < 0.001) and the nonlinear model produced a R = .90 (*P* < 0.001).

For the combined analysis, there was a significant correlation of R = .89 (*P* < 0.001) for the linear model and R = .90 (*P* < 0.001) for the nonlinear model.

##### MP force transducer

Using only the metrics derived from the force transducer, the mean shoulder force (deltaz) produced a moderate correlation of R = .62 (*P* < 0.001) for the linear model and R = .66 (*P* < 0.001) for the nonlinear model. Using the 4 means of the individual shoulder metrics (AB, AD, F, and E) produced a similar result of R = .66 (*P* < 0.001) for the linear models and R = .69 *(P* < 0.001) for the nonlinear model.

#### Correlation between clinical scales

The majority of the clinical scales selected for this study, the FMA-UE, WMFT, and MRC, were highly correlated, except for the BI. See Figs. 1 and 2 for a heatmap representation of the shoulder-elbow and the wrist and forearm results respectively.

## Discussion

This study provides both incremental and novel advances to the methodology of outcome measure correlation studies in stroke research. This is the first study to our knowledge to investigate the value of including comprehensive distal (wrist and forearm) kinematic data and compare the linear and nonlinear correlation models in the outpatient, chronic stroke population. The methods presented here build on previous work by this group investigating the nonlinear correlation model in the inpatient population (Krebs et al., 2014; Agrafiotis et al., 2021) and the linear correlation model in the chronic outpatient population (Bosecker et al., 2010).

Of note, our results reveal higher correlation values with clinical measures using kinematic and kinetic metrics compared to previous studies (see Additional file 2 for optimized neural network parameters.) (Bosecker et al., 2010; Semrau et al., 2013; Krebs et al., 2014; Colombo et al., 2005; Dukelow, 2017; Agrafiotis et al., 2021; Zollo et al., 2011). Numerous factors may have influenced this outcome. One reason may be the baseline characteristics of the patient population (Table 4), which represent a broader more distributed spectrum of patient impairment (FMA-UE of 7–57) than previous studies, which can also be explained from a mathematical perspective (Additional file 1). Bosecker et al. (2010) recruited a large sample of 111 patients in the chronic stage of recovery with an admission FMA-UE of 7–38. Both the FMA-UE and MRC scores were included in their correlation analysis using a linear regression model, but displayed lower R values (FMA-UE = .80 and MRC = .80) than what was found in this study, even for our linear model. Zollo et al. (2011) investigated the correlation of robotic kinematics using data from 15 chronic patients post stroke with an admission FMA-UE of 8–36. This group also used robotic measures to evaluate the correlation with the FMA-UE and MRC using a linear regression method, and reported a moderate R value for the FMA-UE(R = .67) and strong correlation (R = .77) for the MRC. It is well known that the value of a correlation is greater when there is greater variability in the observations studied (Goodwin & Leech, 2006).

Consistent with the results of Bosecker et al. (2010), kinematic submovement characteristics (see Table 2 for metrics and definitions (Krebs et al., 1999)) had little influence on the correlation modeling between kinematics and the clinical measures. The only submovement metric seen to have a R > .5 was submovement duration, which was selected for the BI correlation analyses (which had the lowest correlation value.) Although submovement characteristics had a low impact in both this and Bosecker et al.’s (2010) correlation study, these metrics have been shown to be very important for enhancing our understanding of UE motor recovery (Dipietro et al., 2012; Rohrer et al., 2002).

In keeping with previous findings, the nonlinear model consistently performed better than the linear correlation model for predicting clinical measures (Table 3 and Additional files 1 and 2). (Krebs et al., 2014; Agrafiotis et al., 2021; Krakovska et al., 2019) Our findings contribute to the justification for the convenient use of one (nonlinear) model to predict clinical measures across the continuum of care (acute, sub-acute, and chronic). The variation in nonlinear and linear models of correlation are less dramatic than what is seen in the subacute population, likely due to less variability in the patient presentation. (Krakovska et al., 2019) The use of neural networks and a refined metric selection algorithm was likely influential in the superior correlation results described in this study.

The inclusion of wrist kinematic data is novel within this field of study. Although fitting the wrist data in the linear and nonlinear models showed a strong correlation with the clinical measures, it did not substantially improve the already high correlation provided by the shoulder-elbow data alone. This finding is valuable for numerous reasons. Clinically it is more efficient to only perform one robotic evaluation, therefore the results from this study do not support the need to complete both a wrist and shoulder-elbow robotic evaluation in order to predict the results of clinical measures. The clinician can select the robot that best suits the patient’s impairments and goals without compromising the ability to generate predictions of clinical measures. We also saw that either robot is reliable at predicting the clinical scales in both high and low functioning patients (Table 3).

Correlation among clinical measures was high, except for the BI. The correlation between the FMA-UE and MP (total, R = .94) in this study was higher than what was published to date in the chronic stroke population. Bosecker et al. (2010) reported a strong correlation between MP and both the FMA-UE (.79) and Motor Status Scale (.77, a similar measure to the FMA-UE but consisting of a finer grading scale for UE impairment for the subacute population.) Of note, the study by Bosecker et al. (2010) on chronic stroke survivors differed from this study by using only a linear regression model. Krebs et al. (2014) and Agrafiotis et al. (2021) also reported high correlation with MRC and FMA-UE (R = .93). Therefore the robot metrics were seen to predict the outcome of FMA-UE, WMFT, and MRC consistently.

Of note, the BI analysis in this study indicates that the measure does not correlate well with either the clinical or robotic measures, confirming previous reports. (Chen & Winstein, 2009; Mayo et al., 2002) In a recent large cohort study of 434 subjects in the chronic stage of recovery, Mayo and colleagues (Mayo et al., 2002) reported patients had a mean BI score of 90.6/100, yet 65% of this population could not incorporate their affected UE into ADLs. Sivan et al. (2011) also reported limited responsiveness of the BI involving chronic patients with severe impairments. It appears there are significant variations within and between the results of a patient’s BI score, which implies that a patient’s mood and other factors may influence or bias the scoring of the scale.

It is a common misunderstanding that the relative weight of individual variables in the models and the meaning of the regression model coefficients can be determined. The confidence intervals calculated in our correlation analysis were often large and contained zero, which prompted an investigation into whether groups of metrics used in the model were multicollinear. Multicollinearity occurs when there is a strong correlation among independent variables, often leading to highly significant regression models. Nonetheless information about the individual contributions of each variable cannot be determined. (Freund et al., 2006) It does not affect the fit of a model or the ability to predict point estimates of the response variable. In addition, the validation data indicates how well the model generalizes. A measure of multicollinearity is the variance inflation factor (VIF) and is calculated by completing a linear regression of x_j_ on all other independent variables in the model. The coefficient of determination, R_j2_, for each variable is determined and VIF = [1/(1- R_j2_)]. A common rule of thumb is that a VIF greater than 10 indicates a high level of multicollinearity (corresponding to R_j2_ = 0.9). We found that multiple kinematic and kinetic variables had VIFs above this value indicating that while these models are still valid to calculate clinical scores, the individual contributions of each variable cannot be established.

**Fig. 3:**
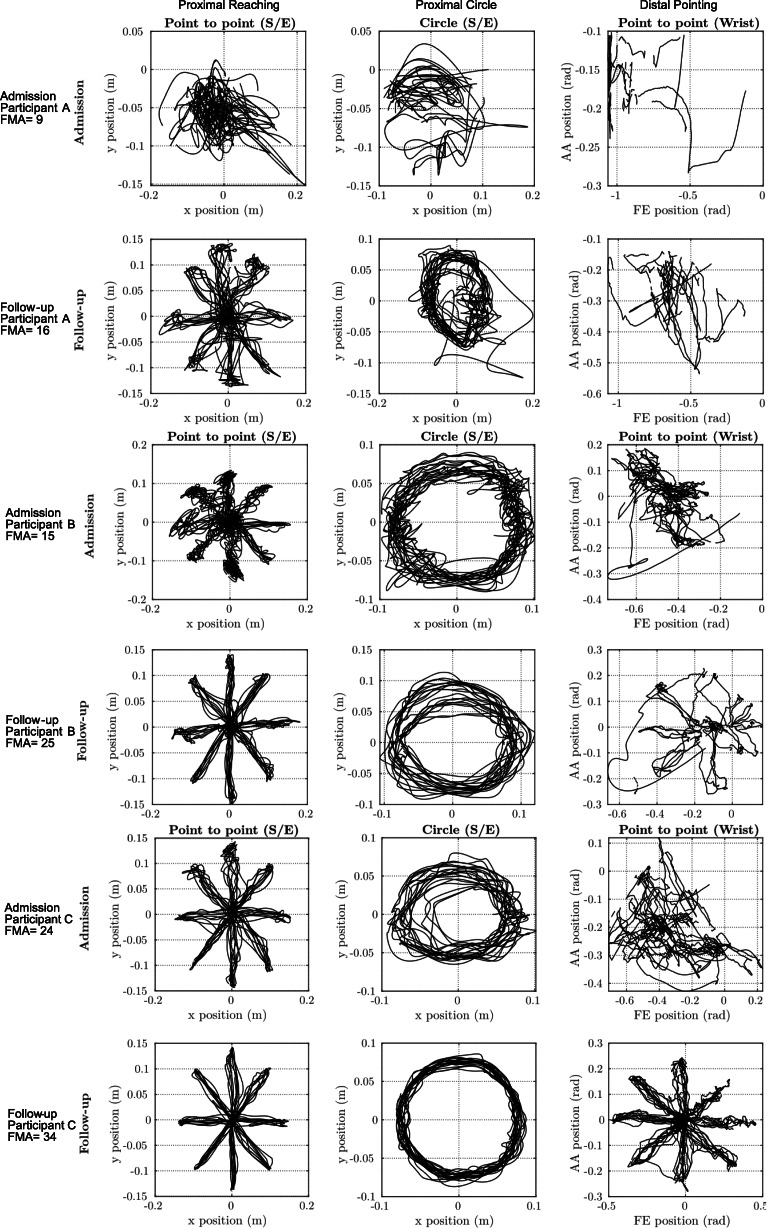
Unassisted proximal and distal movement attempts of three representative stroke participants at study baseline and completion

Although the methodology used in this study led to higher correlations for the clinical measures than previous studies, the results may not apply to all patient populations. It is unclear if the superior correlations generated with the nonlinear model using our methodology extrapolate to other phases of stroke recovery, which warrants future investigation within the acute and subacute population. The same limitation may apply to the strength of wrist kinematic data correlating with clinical measures. Contrary to our expectations, the wrist data did not substantially improve the correlation value compared to the shoulder-elbow result alone. As it appears that the time-course of the wrist recovery is relatively delayed compared to the shoulder-elbow (Fig. 3), we erroneously predicted that it would have had a big impact. It remains to be tested whether the wrist data may be more influential in the acute or subacute stages of post-stroke recovery or milder cases. In addition, instead of training a model that correlates with the total FMA-UE score, it would be of high interest to explore the correlation between the distal wrist robotic data and the corresponding wrist sub-scale of the FMA-UE.

## Conclusions

The improved method of correlation modeling for robotic kinematics and clinical measures in this study is an important step towards objectively assessing and planning a patient’s rehabilitation journey in the chronic stage of stroke recovery. The often time consuming and subjective nature of clinical measures and the growing access and validity of robotic measures will likely increase the demand for reliable correlation models such as the methods described in this study. Robotic measures offer enhanced objectivity of movement quality, accuracy, and standardization that can improve our understanding of the complex process of motor recovery. As this study confirms, robotic kinematics are well suited to representing impairments in body function or structure but are perhaps restricted in their ability to translate kinematic data to performance of ADLs or participation in real-world environments, which warrants further study.

## Supplementary Information

**Additional file 1.**

**Additional file 2.**

## Data Availability

We anticipate that the data captured and created by this research will be of broad interest to communities engaged in research on human motor behavior. Data generated by this research project will be made publicly accessible through a portal linked to our lab homepage http://the77lab.mit.edu/. Access to these data will be “read-only” and password protected. Passwords will be made freely available upon submission of a request by email agreement that the source of the data will be acknowledged in any publication arising from use of these data.

## Abbreviations

tDCS: Transcranial direct current stimulation
UE: Upper extremity
ADLs: Activities of daily living
FMA-UE: Fugl-Meyer Assessment of Upper Extremity Motor Recovery after Stroke
WMFT: Wolf Motor Function Test
BI: Barthel Index
MRC: Medical Research Council Motor Power score
S/E: Shoulder-elbow
F: Flexion
E: Extension
AB: Abduction
AD: Adduction
RD: Radial-deviation
UD: Ulnar deviation
P: Pronation
S: Supination
MP: Motor power
