# Supplementary material for: Robotic Kinematic measures of the arm in chronic Stroke: part 2 – strong correlation with clinical outcome measures
Source: Bioelectron Med. 2021 Dec 29;7:21. doi: 10.1186/s42234-021-00082-8 (PMC8715630; doi:10.1186/s42234-021-00082-8)
Supplement: Supplementary file 1 — Additional file 1. [file 42234_2021_82_MOESM1_ESM.docx]

**Additional file 1: Mathematical perspective on the spread of clinical scores**

Apart from the clinical debate on high predictability of clinical scores from chronic outpatients, it is also necessary to employ a mathematical perspective on the distribution of data points with respect to their respective clinical scores. In this paper we use the correlation between model prediction and the expected output to evaluate the goodness of fit of a model, given by $R^{2}$, defined as follows:

$R^{2}=1-\frac{\frac{1}{n}\sum_{i=1}^{n} {(y_{i}-\hat{y_{i}})}^{2}}{\frac{1}{n}\sum_{i=1}^{n} {(y_{i}-\underline{y})}^{2}}=1-\frac{{VAR}_{Model}}{{VAR}_{Total}}$ A1.

where ${VAR}_{Total}$ is the variance, i.e., mean squared distances between every i-th point $y_{i}$ and the mean $\underline{y}$, whereas ${VAR}_{Model}$ is the mean squared residual with respect to a model fitted to the dataset.

From a linear perspective, for example, we can consider both ${VAR}_{Total}$ and ${VAR}_{Model}$ as mean squared residuals arising, respectively, from a model with no slope (the mean) and a model with a slope adjusted to the data. Therefore, ${VAR}_{Total}$ is always larger because a model non adjusted to data (no slope) yields larger residuals, whereas ${VAR}_{Model}$ yields lower residuals because it is adjusted to the data. That said, the fraction tends to be smaller, as the variance of data points increases. Hence, $R^{2}$ tends to $1$.

In practical terms, a shorter window of clinical scores may yield lower values of $R^{2}$ in comparison to the full range of a scale.
