# Supplementary material for: Robotic Kinematic measures of the arm in chronic Stroke: part 2 – strong correlation with clinical outcome measures
Source: Bioelectron Med. 2021 Dec 29;7:21. doi: 10.1186/s42234-021-00082-8 (PMC8715630; doi:10.1186/s42234-021-00082-8)
Supplement: Supplementary file 2 — Additional file 2. [file 42234_2021_82_MOESM2_ESM.docx]

**Additional file 2: Nonlinear models estimating the FMA-UE, given S/E or S/E and wrist data.**

For both nonlinear models (Additional file 2 Tables 1 and 2), an ensemble of the four models, in terms of mean, must yield the final output.

**Additional file 2, Table 1** - Weights of the ensemble of neural networks that constitute the nonlinear model for the FMA-UE correlation, given S/E data.

| **Layer/node** | **Weight** | **Model 1** | **Model 2** | **Model 3** | **Model 4** |
| --- | --- | --- | --- | --- | --- |
| L2N1 | w0 | 2.651745 | 1.35879 | -2.288135 | -2.623445 |
|  | w1 | 1.705711 | 0.178818 | -2.668738 | 1.231372 |
|  | w2 | 0.196343 | 1.322677 | -0.12641 | -0.67358 |
|  | w3 | -0.025334 | -0.208155 | 0.401209 | -0.224087 |
|  | w4 | -2.059048 | 0.651006 | 1.131175 | 1.364455 |
|  | w5 | -0.169044 | -1.014909 | 0.328886 | -1.796512 |
|  | w6 | -0.522972 | 0.544617 | 0.817448 | -0.33786 |
| L2N2 | w0 | 0.209553 | -3.869454 | -2.525723 | -1.880432 |
|  | w1 | 0.066768 | -0.855441 | 0.893196 | -2.088235 |
|  | w2 | 0.702611 | -0.128405 | -0.075536 | 0.038249 |
|  | w3 | -0.884474 | -0.065442 | -0.597529 | 0.201751 |
|  | w4 | 0.841676 | 1.42178 | 0.883846 | 1.057567 |
|  | w5 | -0.757341 | -1.417613 | -0.720293 | 0.442159 |
|  | w6 | 0.289868 | 0.302277 | -0.53256 | 0.835733 |
| L3N1 | w0 | 0.458473 | 1.156568 | 5.558105 | 1.241911 |
|  | w1 | -1.058998 | 0.206183 | 1.041446 | 1.055794 |
|  | w2 | 0.346208 | 2.015478 | 5.429382 | 1.104092 |

**Additional file 2, Table 2** - Weights of the ensemble of neural networks that constitute the nonlinear model for the FMA-UE correlation, given S/E and wrist data.

| **Layer/node** | **Weight** | **Model 1** | **Model 2** | **Model 3** | **Model 4** |
| --- | --- | --- | --- | --- | --- |
| L2N1 | w0 | 0.100405 | -0.499624 | 0.917564 | 0.28733 |
|  | w1 | 0.412031 | -0.64457 | -0.910925 | 0.089558 |
|  | w2 | -0.256844 | -0.515516 | 0.244053 | -0.012349 |
|  | w3 | 0.35973 | 0.291215 | -0.495911 | 0.25471 |
|  | w4 | 0.224924 | -0.271208 | 0.197361 | -0.032417 |
|  | w5 | -0.610725 | 0.488541 | 1.05291 | -0.033304 |
|  | w6 | -0.125555 | -0.297185 | -1.170956 | -0.01999 |
|  | w7 | 1.204007 | 0.564216 | 0.451516 | 0.403285 |
|  | w8 | -0.29505 | 0.090108 | -0.376013 | -0.124665 |
|  | w9 | -0.654584 | -0.47142 | 0.276252 | -0.19922 |
|  | w10 | 0.464651 | 0.788101 | 0.134621 | 0.363913 |
|  | w11 | -0.29234 | -0.168995 | -0.22671 | -0.002602 |
| L2N2 | w0 | 0.675229 | -1.719442 | -0.220476 | 3.099416 |
|  | w1 | 0.628326 | -0.59014 | 0.184195 | -1.748653 |
|  | w2 | 0.450943 | 0.436204 | 0.367614 | -0.897005 |
|  | w3 | -0.306028 | -0.832092 | -0.23557 | 1.395623 |
|  | w4 | 0.905761 | 0.254605 | 0.032158 | 0.893904 |
|  | w5 | -0.537267 | -0.044102 | 0.039324 | 1.244433 |
|  | w6 | 0.042878 | 0.246911 | -0.028137 | -1.058052 |
|  | w7 | -0.140713 | -1.50755 | -0.409774 | 0.778307 |
|  | w8 | 0.132989 | 1.317929 | -0.142964 | -0.593424 |
|  | w9 | 0.214891 | 1.315244 | 0.566939 | -1.37962 |
|  | w10 | -0.636054 | -0.210806 | -0.582138 | -1.72326 |
|  | w11 | -0.242302 | 0.231229 | -0.271664 | 0.015366 |
| L3N1 | w0 | -0.123029 | -0.104083 | 0.19395 | 0.444519 |
|  | w1 | -0.617778 | -0.491291 | -0.248219 | -1.123216 |
|  | w2 | 0.263766 | 0.375458 | 0.826389 | -0.398329 |

Note: S/E= shoulder-elbow, FMA-UE= Fugl-Meyer Assessment of Upper Extremity Motor Recovery after Stroke.
